# Transcriptomic Alterations in Peripheral Blood of Patients with Metabolic Syndrome

**DOI:** 10.3390/ijms27219564

**Published:** 2026-10-26

**Authors:** Alpay Medetalibeyoglu, Yasemin Oyaci, Naci Senkal, Sevde Sayin, Merve Guzel Dirim, Hilal Senkal, Mustafa Pehlivan, Tufan Tukek, Sacide Pehlivan

**Affiliations:** 1 Department of Internal Medicine, Acıbadem Altunizade Hospital, Istanbul 34718, Türkiye; 2 Department of Basic Sciences, Istanbul Nisantası University, Istanbul 34398, Türkiye; yaseminf@hotmail.com; 3 Department of Internal Medicine, Istanbul Faculty of Medicine, Istanbul University, Istanbul 34093, Türkiye; 4 Department of Medical Biology, Istanbul Faculty of Medicine, Istanbul University, Istanbul 34093, Türkiyesacide.pehlivan@istanbul.edu.tr (S.P.); 5 Department of Internal Medicine, Bahcelievler State Hospital, Istanbul 34186, Türkiye; drmerveguzel@gmail.com; 6 Department of Internal Medicine, Bagcilar Training and Research Hospital, Istanbul 34200, Türkiye; 7 Department of Hematology, Basaksehir Cam and Sakura City Hospital, Istanbul 33480, Türkiye; psacide@hotmail.com

**Keywords:** metabolic syndrome, RNA-seq, transcriptomics, gene expression, RT-qPCR evaluation

## Abstract

Metabolic syndrome (MetS) is a complex cardiometabolic disorder characterized by interconnected abnormalities in glucose and lipid metabolism, adiposity, and vascular regulation. Transcriptomic profiling may provide insight into molecular alterations associated with MetS. This study aimed to identify peripheral blood gene expression changes in MetS using RNA sequencing and to evaluate selected candidate genes by RT-qPCR in an independent cohort. RNA sequencing was performed in 15 patients with MetS and 10 healthy controls; after exclusion of three MetS samples based on PCA, differential expression analysis included 12 patients with MetS and 10 controls. Six candidate genes (*FKBP1B*, *PLIN2*, *ARVCF*, *NAA40*, *ALOX15B*, and *MUSTN1*) were subsequently evaluated by RT-qPCR in an independent cohort of 146 patients with MetS and 116 controls. Relative expression changes were calculated using the 2^−ΔΔCt method, and multiple testing was controlled using the Benjamini–Hochberg false discovery rate (FDR) procedure. Among 19,668 protein-coding genes, 730 showed nominal differential expression (*p* < 0.05), whereas only *FKBP1B* and *EVA1C* remained significant after FDR correction. In the RT-qPCR cohort, *FKBP1B*, *ARVCF*, and *NAA40* showed significantly higher expression in MetS, corresponding to 6.33-, 1.87-, and 2.34-fold increases, respectively; all remained significant after FDR correction. No significant differences were observed for *PLIN2*, *ALOX15B*, or *MUSTN1*. Exploratory analyses identified no robust associations between candidate-gene expression and metabolic or cardiovascular clinical parameters after multiple-testing correction. FKBP1B remained significant after transcriptome-wide FDR correction and showed concordant upregulation in the independent RT-qPCR cohort, while the nominal RNA-seq candidates ARVCF and NAA40 also showed significantly increased expression by RT-qPCR. These findings identify transcriptional alterations associated with MetS and warrant further validation in independent cohorts and functional studies.

## 1. Introduction

Metabolic syndrome (MetS) is a complex cardiometabolic disorder characterized by the clustering of interrelated abnormalities, including central obesity, insulin resistance, hypertension, hypertriglyceridemia, reduced high-density lipoprotein cholesterol levels, and impaired glucose regulation. Its prevalence has increased substantially worldwide, with estimates indicating that approximately one-quarter of the global population is affected [1]. Given its strong association with type 2 diabetes mellitus (T2DM), cardiovascular disease, and other chronic complications, MetS represents an important global public health burden.

Genome-wide association studies have identified numerous genetic loci associated with metabolic traits and related diseases [2]. However, translating these associations into biological mechanisms remains challenging because many risk loci do not directly reveal the causal genes, relevant tissues, or downstream molecular pathways involved. Gene expression profiling offers a complementary approach by capturing transcriptional alterations that may reflect the functional consequences of metabolic dysregulation and help clarify molecular processes underlying MetS.

Previous transcriptomic and molecular studies have implicated pathways related to energy metabolism, inflammation, lipid handling, glucose homeostasis, adipose tissue function, oxidative stress, and mitochondrial regulation in MetS [3]. These alterations are thought to contribute to chronic low-grade inflammation, impaired insulin signaling, abnormal lipid accumulation, and systemic metabolic imbalance. Nevertheless, the transcriptional landscape of MetS remains incompletely characterized, and findings derived from small discovery cohorts require validation in larger independent cohorts.

In the present study, we performed RNA sequencing (RNA-seq) in peripheral blood samples from patients with MetS and healthy controls to identify transcriptional alterations associated with the syndrome. Selected genes from the RNA-seq analysis were subsequently assessed by RT-qPCR in a larger independent cohort, and their expression levels were examined in relation to major metabolic and cardiovascular clinical parameters. This combined approach was designed to identify peripheral blood transcriptional signals associated with MetS.

## 2. Results

Baseline demographic, clinical, metabolic, and medication characteristics of the RNA-seq discovery and RT-qPCR evaluation cohorts are presented in Supplementary Table S1. Three MetS samples showed clear separation from the remaining samples in the PCA and were excluded from downstream analysis. Differential expression analysis was therefore performed using 12 MetS samples and 10 healthy controls.

RNA-seq analysis evaluated 19,668 protein-coding genes. Using the exploratory criteria of nominal *p* < 0.05 and a linear fold change >1.2 or <1/1.2, 730 protein-coding genes were classified as differentially expressed, including 519 upregulated and 211 downregulated genes. After Benjamini–Hochberg FDR correction, only *FKBP1B* (log2FC = 1.05; linear fold change = 2.07; FDR-adjusted *p* = 0.019) and *EVA1C* (log2FC = 0.54; linear fold change = 1.45; FDR-adjusted *p* = 0.019) remained statistically significant.

**Figure 1:**
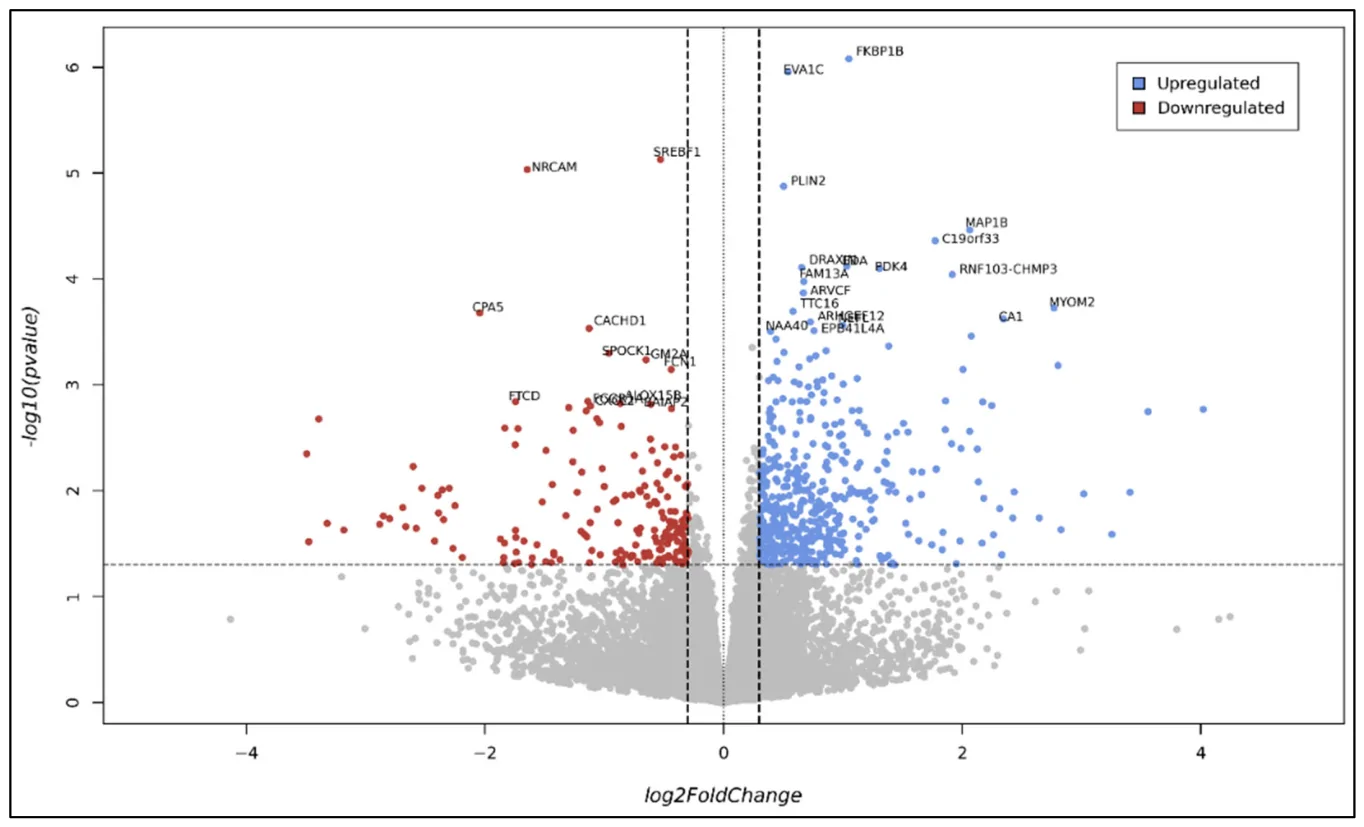
Volcano plot of differential gene expression between patients with metabolic syndrome and healthy controls. Each point represents a protein-coding gene, plotted according to its log2 fold change and −log10 (*p*-value). Genes with a linear fold change >1.2 and *p* < 0.05 were classified as upregulated and are shown in blue, whereas genes with a linear fold change <1/1.2 and *p* < 0.05 were classified as downregulated and are shown in red; the remaining genes are shown in gray. Selected top-ranked genes are labeled. Positive log2 fold-change values indicate higher expression in MetS, whereas negative values indicate lower expression.

**Figure 2:**
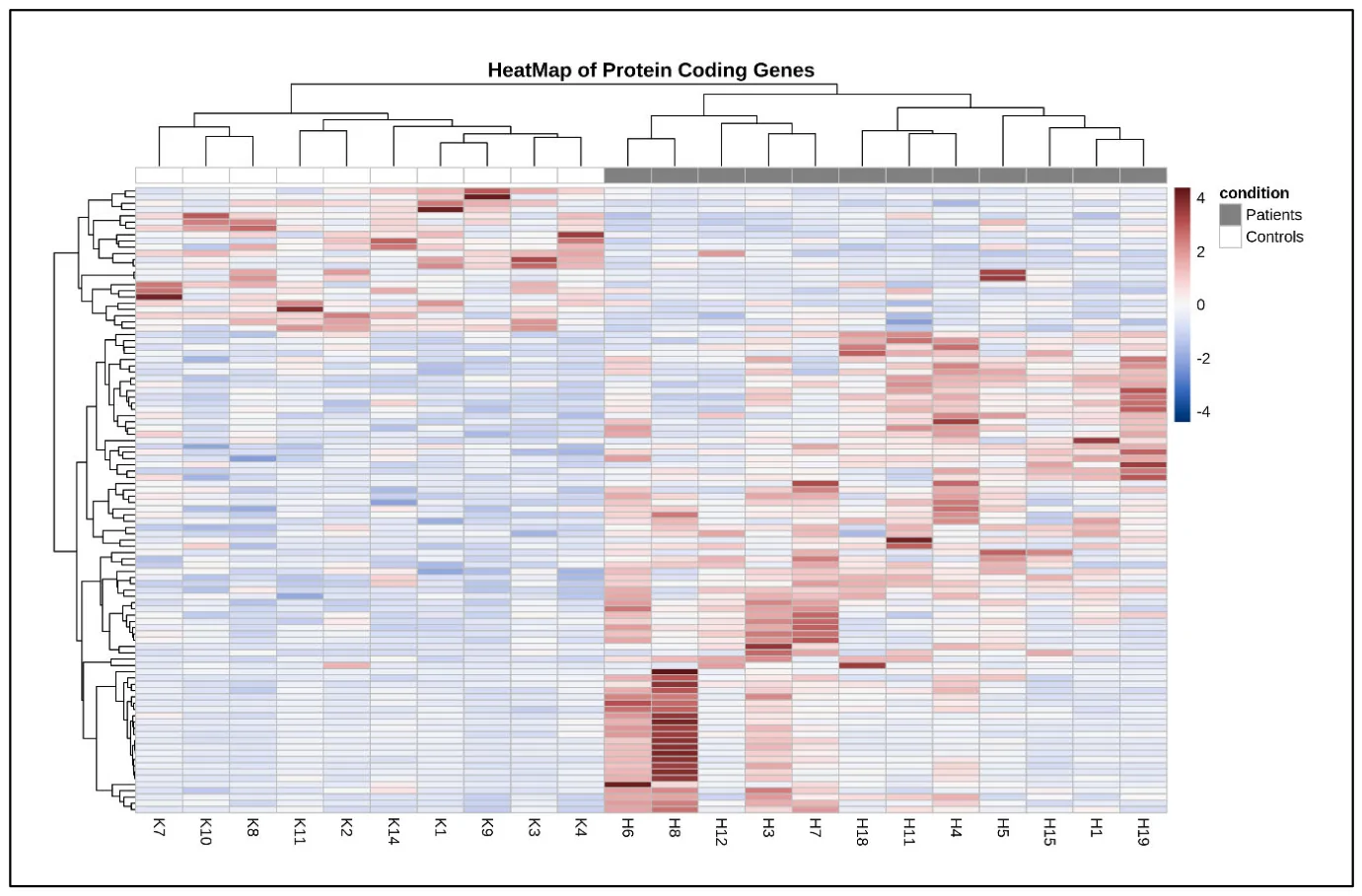
Heatmap of the top 100 protein-coding genes ranked by nominal *p*-value in patients with metabolic syndrome and healthy controls. Hierarchical clustering was applied to these genes and samples. Pearson correlation was used as the distance metric for clustering, and each row was autoscaled for heatmap visualization. The color scale represents standardized relative expression levels, with red indicating higher and blue indicating lower expression relative to the gene-specific mean. Sample labels beginning with H denote patients with metabolic syndrome, whereas labels beginning with K denote healthy controls.

The overall differential expression pattern was visualized using a volcano plot (Figure 1), whereas the heatmap illustrates the expression profiles of selected top-ranked genes across individual samples (Figure 2). The complete list of all 730 nominally differentially expressed protein-coding genes (nominal *p* < 0.05), comprising 519 upregulated and 211 downregulated genes, is provided in the Supplementary Data S1.

Functional enrichment analysis identified significant terms predominantly within Gene Ontology categories, with additional significant findings in Reactome and WikiPathways analyses, whereas no KEGG pathway reached statistical significance under the applied criteria. Candidate-focused examination of the GO results showed that several of the RT-qPCR-selected genes contributed to significantly enriched functional categories. In particular, ARVCF was represented in cell–cell adhesion and cell-junction-related terms, while ARVCF, FKBP1B, ALOX15B, and MUSTN1 contributed to the enriched developmental-process category. Complete enrichment results are provided in the Supplementary Data S2. GSEA of the full ranked protein-coding dataset did not identify any Hallmark gene set that remained significant after FDR correction. Among the strongest absolute enrichment signals were observed for heme metabolism, Notch signaling, WNT/β-catenin signaling, and Hedgehog signaling; however, these findings did not survive multiple-testing correction and were therefore considered exploratory.

Among the genes showing differential expression in the RNA-seq analysis, six candidate genes were selected for targeted RT-qPCR evaluation based on their RNA-seq ranking, direction of expression change, and biological relevance. *FKBP1B*, *PLIN2*, *ARVCF*, and *NAA40* were selected from the upregulated genes, whereas *ALOX15B* and *MUSTN1* were selected from the downregulated genes. The RNA-seq log2 fold changes, nominal and FDR-adjusted *p*-values, and direction-specific rankings of the six selected genes are presented in Supplementary Table S2.

**Figure 3:**
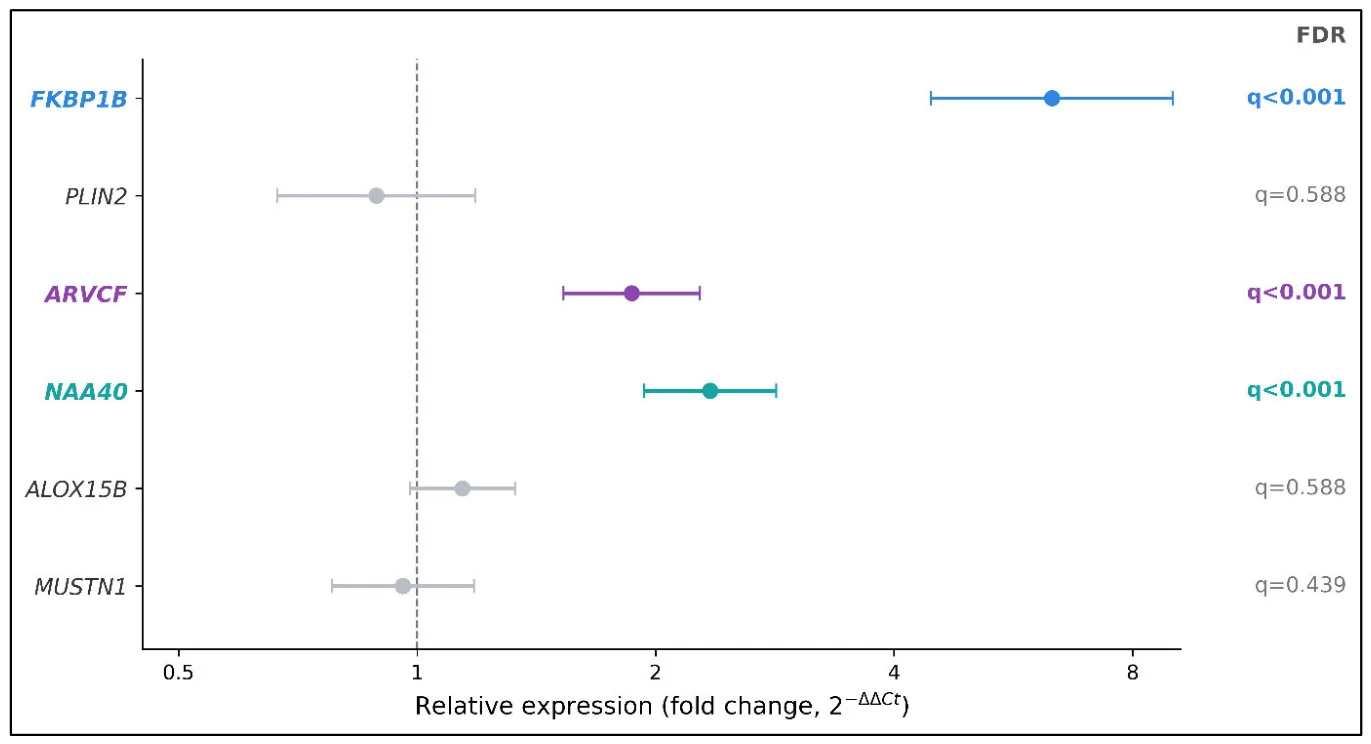
RT-qPCR evaluation of selected candidate genes in metabolic syndrome. Relative expression levels of *FKBP1B*, *PLIN2*, *ARVCF*, *NAA40*, *ALOX15B*, and *MUSTN1* in patients with metabolic syndrome (MetS) compared with healthy controls are shown as fold changes calculated using the 2^−ΔΔCt method, with the control group used as the calibrator (control = 1). Dots represent fold-change estimates and horizontal lines indicate 95% confidence intervals obtained using bias-corrected and accelerated (BCa) nonparametric bootstrap resampling of ΔCt values and subsequent back-transformation to the fold-change scale. Statistical comparisons between groups were performed using ΔCt values with the Mann–Whitney U test, and *p*-values were adjusted for multiple testing using the Benjamini–Hochberg false discovery rate (FDR) method. *FKBP1B, ARVCF,* and *NAA40* showed significantly higher expression in MetS after FDR correction, whereas *PLIN2, ALOX15B,* and *MUSTN1* showed no significant between-group differences.

RT-qPCR analysis in the larger cohort showed significantly higher expression of *FKBP1B*, *ARVCF*, and *NAA40* in patients with MetS than in healthy controls, corresponding to 6.33-, 1.87-, and 2.34-fold increases, respectively. These differences remained statistically significant after Benjamini–Hochberg FDR correction. In contrast, no significant between-group differences were observed for *PLIN2*, *ALOX15B*, or *MUSTN1*. The relative expression changes and corresponding 95% confidence intervals are shown in Figure 3.

Exploratory analyses were performed to examine the relationships between candidate-gene expression and selected metabolic and cardiovascular clinical parameters. Among the continuous variables, only *FKBP1B* ΔCt values showed a nominal correlation with HDL-C (Spearman ρ = −0.187, *p* = 0.032); however, this association did not remain significant after FDR correction (FDR-adjusted *p* = 0.997). No other nominally significant correlations were observed, and none of the gene–clinical parameter associations remained significant after FDR correction (Supplementary Table S3). Similarly, candidate-gene expression did not differ significantly according to hypertension or ischemic heart disease status after FDR correction (Supplementary Table S4).

## 3. Discussion

MetS is characterized by a complex interplay of metabolic, inflammatory, and vascular disturbances, and transcriptomic profiling may provide insight into the molecular alterations underlying this heterogeneity. In the present study, RNA-seq identified 730 genes with nominal differential expression in peripheral blood, although only FKBP1B and EVA1C remained significant after correction for multiple testing. Targeted RT-qPCR analysis in a larger independent cohort further demonstrated significantly increased expression of *FKBP1B*, *ARVCF*, and *NAA40* in patients with MetS, whereas *PLIN2*, *ALOX15B*, and *MUSTN1* showed no significant between-group differences. Among these findings, *FKBP1B* showed the strongest evidence, remaining significant after transcriptome-wide FDR correction and showing concordant upregulation by RT-qPCR. *ARVCF* and *NAA40* were nominal RNA-seq findings but were also significantly upregulated in the independent RT-qPCR cohort. The absence of robust associations with individual clinical parameters suggests that these expression changes may reflect broader features of the MetS phenotype rather than specific cardiometabolic components.

FKBP1B, also known as FKBP12.6, is a member of the FK506-binding protein family and is involved in intracellular calcium regulation and cellular signaling. Members of the FKBP family participate in several signaling pathways involved in cellular homeostasis [4]. In pancreatic islets, *Fkbp1b* expression has been reported to decrease during streptozotocin-induced β-cell damage, together with other genes involved in β-cell function and diabetes-related pathways [5]. These observations suggest that altered FKBP1B expression may accompany disturbances in β-cell homeostasis, although its specific role in human metabolic disease remains incompletely understood.

In the present study, *FKBP1B* was one of only two genes that remained significant after FDR correction in the RNA-seq discovery analysis and showed a concordant 6.33-fold increase in the larger RT-qPCR cohort. This consistency across two analytical platforms strengthens the evidence that increased peripheral blood *FKBP1B* expression is associated with MetS. A nominal association was observed between FKBP1B ΔCt values and HDL-C (Spearman ρ = −0.187, *p* = 0.032); however, this association did not remain significant after FDR correction (FDR-adjusted *p* = 0.997), and no other significant associations were identified with the evaluated clinical parameters. Although previous experimental work has linked altered FKBP1B-related signaling to disturbances in calcium homeostasis and β-cell function, the increased expression observed in peripheral blood in our study may reflect a distinct systemic transcriptional response associated with MetS. However, the present findings do not establish whether altered FKBP1B expression contributes to disease pathogenesis or occurs as a secondary consequence of the metabolic phenotype. Further studies are needed to determine the cellular origin and functional significance of increased *FKBP1B* expression in MetS.

PLIN2 encodes perilipin-2, a lipid droplet-associated protein involved in intracellular lipid storage and cellular energy metabolism [6]. Experimental evidence has implicated PLIN2 in lipid droplet dynamics, adipocyte lipid handling, and metabolic regulation. PLIN2 has been shown to play a positive role in adipocyte lipolysis [7], whereas PLIN2 deficiency in animal models has been associated with protection against diet-induced obesity, reduced adipose tissue inflammation, and attenuation of hepatic steatosis [8]. PLIN2 has also been implicated in the regulation of hepatic lipid droplet morphology and triglyceride storage under fasting conditions [9]. Collectively, these findings support a role for PLIN2 in lipid homeostasis, although its effects appear to be tissue- and context-dependent.

In the present study, *PLIN2* was identified among the nominally upregulated genes in the RNA-seq discovery analysis; however, this finding was not reproduced in the larger RT-qPCR cohort, in which no significant difference in *PLIN2* expression was observed between patients with MetS and healthy controls. In addition, no significant associations were observed between *PLIN2* expression and the evaluated metabolic or cardiovascular clinical parameters. These findings suggest that the initial RNA-seq signal for *PLIN2* may represent a discovery-stage observation rather than a robust peripheral blood expression alteration in MetS. Given the established role of PLIN2 in lipid droplet biology and metabolic regulation, further studies in larger cohorts and metabolically active tissues may help clarify whether its relevance to MetS is tissue-specific or context-dependent.

ARVCF encodes armadillo repeat protein deleted in velo-cardio-facial syndrome, a member of the p120-catenin subfamily involved in cadherin-associated cell–cell adhesion and adherens junction organization [10]. ARVCF has been shown to contribute to the stability of cadherin-based junctional complexes and tissue organization [10]. In addition, genetic and experimental studies have implicated ARVCF in neurobehavioral processes, including schizophrenia susceptibility [11] and dopaminergic signaling involved in nicotine reward [12]. However, its biological role in metabolic disorders remains largely unexplored.

In the present study, *ARVCF* was nominally upregulated in the RNA-seq discovery analysis, and this direction of change was reproduced in the larger RT-qPCR cohort, in which *ARVCF* expression was 1.87-fold higher in patients with MetS than in healthy controls and remained significant after FDR correction. No significant associations were observed between *ARVCF* expression and the evaluated metabolic or cardiovascular clinical parameters. Although ARVCF has not previously been directly linked to MetS, its established role in cadherin-associated junctional regulation provides a potential biological context for the observed expression change. However, the relevance of ARVCF to MetS remains uncertain and requires mechanistic and tissue-specific investigation.

NAA40 encodes N-α-acetyltransferase 40, an acetyltransferase responsible for the N-terminal acetylation of histones H2A and H4 [13]. Through its role in histone acetylation, NAA40 has been implicated in epigenetic regulation, chromatin organization, and transcriptional control. Emerging evidence also links NAA40 to metabolic processes. Experimental studies have shown that NAA40 modulates cellular acetyl-CoA levels, lipid synthesis, and insulin signaling, with NAA40 expression also associated with insulin sensitivity in obesity [14]. In addition, loss of Nat4/NAA40-mediated histone acetylation has been linked to calorie restriction-associated longevity in experimental models [15].

In the present study, NAA40 was nominally upregulated in the RNA-seq discovery analysis, and this direction of change was reproduced in the larger RT-qPCR cohort, in which NAA40 expression was 2.34-fold higher in patients with MetS than in healthy controls and remained significant after FDR correction. No significant associations were observed between *NAA40* expression and the evaluated metabolic or cardiovascular clinical parameters. Given the reported links between NAA40, acetyl-CoA metabolism, lipid synthesis, and insulin signaling, the observed increase in peripheral blood NAA40 expression may be related to metabolic alterations associated with MetS. However, its functional relevance in MetS remains uncertain and requires mechanistic and tissue-specific studies.

ALOX15B encodes arachidonate 15-lipoxygenase type B (15-LOX-2), a member of the lipoxygenase family that catalyzes the oxygenation of polyunsaturated fatty acids and contributes to the generation of bioactive lipid mediators [16]. ALOX15B has been implicated in inflammatory processes and lipid homeostasis [16]. Evidence from studies of related lipoxygenase pathways, particularly ALOX15/12–15-lipoxygenase, has further demonstrated links with diabetes-related inflammation, obesity-associated inflammatory responses, and insulin resistance [17,18,19]. Although these findings do not directly establish a metabolic role for ALOX15B, they provide biological context for investigating lipoxygenase-related pathways in MetS.

In the present study, *ALOX15B* was identified as a nominally downregulated gene in the RNA-seq discovery analysis; however, this finding was not reproduced in the larger RT-qPCR cohort, in which no significant difference in *ALOX15B* expression was observed between patients with MetS and healthy controls. No significant associations were observed between *ALOX15B* expression and the evaluated metabolic or cardiovascular clinical parameters. Thus, the RNA-seq signal for *ALOX15B* should be regarded as a discovery-stage observation rather than a reproducible peripheral blood expression alteration in MetS. Given the involvement of lipoxygenase pathways in lipid mediator biology and metabolic inflammation, further studies may clarify whether ALOX15B has tissue-specific or context-dependent relevance to MetS.

MUSTN1 encodes musculoskeletal embryonic nuclear protein 1, a protein implicated in skeletal muscle development and, more recently, adipogenic regulation [20]. Experimental evidence suggests that MUSTN1 may also contribute to metabolic homeostasis. Skeletal muscle-specific ablation of *Mustn1* has been associated with improved glucose tolerance and increased GLUT expression in mice [21]. In addition, MUSTN1 has been shown to promote preadipocyte proliferation, adipogenic differentiation, and lipid deposition, whereas MUSTN1 deficiency protected mice against high-fat diet-induced obesity, hepatic steatosis, and insulin resistance [20]. These findings suggest a potential role for MUSTN1 in metabolic regulation, although the available evidence is predominantly derived from animal and tissue-specific models.

In the present study, *MUSTN1* was identified as a nominally downregulated candidate in the RNA-seq discovery analysis; however, this finding was not reproduced in the larger RT-qPCR cohort, in which no significant difference in *MUSTN1* expression was observed between patients with MetS and healthy controls. No significant associations were observed between *MUSTN1* expression and the evaluated metabolic or cardiovascular clinical parameters. Thus, the RNA-seq signal for *MUSTN1* should be interpreted as a discovery-stage observation rather than a reproducible peripheral blood expression alteration in MetS. Given the experimental evidence linking MUSTN1 to skeletal muscle and adipose tissue metabolism, further tissue-specific and functional studies may clarify whether its relevance to MetS is context-dependent.

### Strengths and Limitations

This study has several strengths. The integration of RNA-seq discovery with RT-qPCR evaluation in an independent and substantially larger cohort strengthened the assessment of whether transcriptomic signals identified at the discovery stage could be reproduced using a separate analytical platform and participant set. This approach identified concordant expression changes for *FKBP1B*, *ARVCF*, and *NAA40*, while also distinguishing RNA-seq findings that were not reproduced by RT-qPCR. In addition, the evaluation of candidate-gene expression in relation to metabolic and cardiovascular clinical parameters provided a broader assessment of the potential clinical relevance of the observed transcriptional alterations.

Several limitations should also be considered. The RNA-seq discovery analysis was based on a relatively small sample, particularly after exclusion of three MetS samples following PCA, which limits statistical power for transcriptome-wide inference and may contribute to discovery-stage findings that are not reproduced in larger cohorts. Gene expression was assessed in peripheral blood; therefore, the observed transcriptional patterns may not reflect those in metabolically active tissues such as liver, adipose tissue, skeletal muscle, or vascular tissue. In addition, demographic and clinical covariates and peripheral blood-cell composition were not incorporated into the RNA-seq model and should therefore be considered when interpreting the discovery-stage transcriptional findings. Moreover, the cross-sectional observational design does not allow us to determine whether the observed transcriptional alterations contribute to MetS pathogenesis or represent secondary responses to the metabolic and inflammatory disturbances associated with the syndrome. Tissue-specific, longitudinal, and functional studies are needed to clarify the biological significance and temporal relationship of these expression changes with MetS. Although EVA1C was one of only two genes that remained significant after transcriptome-wide FDR correction, it could not be independently evaluated by RT-qPCR because reliable and specific amplification could not be achieved despite testing alternative primer pairs. Consequently, one of the strongest discovery-stage signals remains without independent technical evaluation, which limits the completeness of the RNA-seq-to-RT-qPCR evidence chain. EVA1C should therefore be regarded as an FDR-significant discovery finding rather than a validated transcriptional marker. Future studies using redesigned assays or alternative validation platforms should specifically evaluate EVA1C in independent cohorts.

## 4. Methods

### 4.1. Study Population

The study included two independent cohorts. The RNA-seq discovery cohort comprised 15 patients with metabolic syndrome (MetS) and 10 healthy controls, whereas the RT-qPCR evaluation cohort comprised 146 patients with MetS and 116 healthy controls. The two cohorts consisted of distinct, non-overlapping participants recruited from the Internal Medicine Clinic of Istanbul University, Istanbul Faculty of Medicine. The same diagnostic and eligibility criteria were applied to both cohorts. MetS was defined according to the revised National Cholesterol Education Program Adult Treatment Panel III (NCEP ATP III) criteria. Participants were classified as having MetS if at least three of the following five criteria were present: increased waist circumference (>102 cm in men and >88 cm in women), triglyceride levels ≥ 150 mg/dL, reduced high-density lipoprotein cholesterol (HDL-C) levels (<40 mg/dL in men and <50 mg/dL in women), elevated blood pressure (systolic blood pressure ≥ 130 mmHg and/or diastolic blood pressure ≥ 85 mmHg), and fasting blood glucose ≥ 100 mg/dL.

The control group consisted of individuals without a diagnosis of MetS or any known chronic disease and without metabolic abnormalities based on the parameters evaluated in this study. Participants with endocrine disorders, liver or kidney disease, malignancy, pregnancy, active infection, inflammatory disease, or other chronic systemic conditions that could potentially influence gene expression were excluded.

### 4.2. RNA Isolation, Library Preparation, and RNA Sequencing

For the RNA-seq discovery phase, peripheral blood samples were obtained from 15 patients with metabolic syndrome (MetS) and 10 healthy controls. Total RNA was isolated using the TIANGEN RNASimple Total RNA Isolation Kit, TIANGEN Biotech, Beijing, China (Cat. No. 4992858) according to the manufacturer’s instructions. For library preparation, 550 ng of total RNA per sample was subjected to ribosomal RNA depletion using the Illumina Ribo-Zero Plus rRNA Depletion Kit (Illumina, San Diego, CA, USA). The depleted RNA was fragmented and used for first- and second-strand cDNA synthesis, followed by adaptor ligation and library amplification according to the manufacturer’s protocol. Library fragment size was assessed using the QIAxcel Advanced system, and library concentration was measured using the Qubit dsDNA HS Assay Kit. The expected library fragment size was approximately 375–475 bp. Library molarity was calculated based on fragment size and concentration measurements. The libraries were subsequently diluted to the required concentration, pooled, and sequenced on the Illumina NovaSeq 6000 platform using a paired-end 2 × 150-bp sequencing configuration.

### 4.3. Bioinformatic Analysis of RNA-Seq Data

Raw FASTQ files generated by RNA sequencing were subjected to quality control and preprocessing. Adapter trimming and quality filtering were performed using fastp v0.23.2. Read quality before and after preprocessing was assessed using FastQC v0.11.9, and quality-control reports were summarized using MultiQC v1.12. The mean number of paired reads per sample was 40.45 million before trimming and 39.58 million after trimming. Following adapter trimming and quality filtering, the median number of paired reads per sample was 40.46 million (range, 8.52–68.25 million). Q20 was 100% across the processed samples, and the median Q30 score was 99.42% (range, 99.25–99.65%). The median proportion of reads retained after preprocessing was 97.68%, and GC content ranged from 40% to 46%.

High-quality reads were aligned to the human reference genome (GRCh38/hg38) using STAR v2.7.10. Genome annotation was based on GENCODE release 34, and the corresponding GTF annotation file was used for gene-level quantification. The resulting alignment files were processed and indexed using SAMtools v1.15 and Sambamba v0.8.2. Gene-level read counts were generated from the aligned reads using featureCounts implemented in the Subread package v2.0.1. No additional minimum-count threshold was applied to prefilter low-count genes prior to differential expression analysis.

Principal component analysis (PCA) was used to evaluate overall sample clustering and identify samples with markedly divergent global expression profiles. Three MetS samples showed clear separation from the main sample cluster and were excluded from the primary differential expression analysis because of their pronounced divergence in overall transcriptomic profiles. This exclusion was determined at the sample-level assessment stage, prior to differential expression-based candidate-gene selection. Differential expression analysis was consequently performed using 12 MetS samples and 10 healthy controls.

Differential gene expression analysis was conducted in R using DESeq2 v1.34.0 with a single-factor design including group (MetS versus healthy control) as the sole explanatory variable (design = ~ group). Age, sex, BMI, medication use, and other clinical variables were not included as covariates in the DESeq2 model. Given the limited size of the RNA-seq discovery cohort, additional clinical covariates were not incorporated to avoid overparameterization of the discovery-stage model. Sequencing-quality metrics and overall sample clustering by PCA were reviewed for potential technical variation; however, no separate batch term or other technical covariates were included in the DESeq2 model. No additional shrinkage of log2 fold-change estimates was applied. Multiple-testing correction was performed using the Benjamini–Hochberg false discovery rate (FDR) procedure. Genes with a nominal *p*-value < 0.05 were considered in the exploratory differential expression analysis, whereas genes with an FDR-adjusted *p*-value <0.05 were considered statistically significant after correction for multiple testing. PCA and differential expression results were visualized using DEBrowser and custom R scripts.

Functional enrichment analysis was performed using Gene Ontology (GO), KEGG, Reactome, WikiPathways, and Human Phenotype Ontology resources. Enrichment analyses were evaluated separately for the complete, upregulated, and downregulated gene sets, and multiple-testing correction was applied to the enrichment results. Gene set enrichment analysis (GSEA) was also performed using the full ranked protein-coding expression dataset and the MSigDB Hallmark gene set collection. Genes were ranked using the Welch t statistic calculated from log2-transformed normalized expression values. Normalized enrichment scores (NES), nominal *p*-values, and FDR q-values were calculated.

### 4.4. RT-qPCR Evaluation of Selected Candidate Genes

To evaluate selected transcriptomic findings in a larger independent cohort, 146 patients with MetS and 116 healthy controls were analyzed by RT-qPCR.

Candidate-gene selection was completed before RT-qPCR testing and was based exclusively on the RNA-seq results. Among genes showing nominal differential expression, candidates were selected by considering their statistical ranking, direction and magnitude of expression change, and biological relevance to metabolic regulation, lipid metabolism, cellular signaling, and related cardiometabolic processes. To represent both directions of transcriptional change, four upregulated genes (FKBP1B, PLIN2, ARVCF, and NAA40) and two downregulated genes (ALOX15B and MUSTN1) were selected for RT-qPCR evaluation. Candidate-gene selection was not based on absolute log2FC magnitude alone; statistical ranking, direction and magnitude of expression change, and biological relevance were considered jointly. PPI network or hub-gene metrics were not used as criteria in the original candidate-gene selection procedure.

Accordingly, the expression levels of six candidate genes—*FKBP1B*, *PLIN2*, *ARVCF*, *NAA40*, *ALOX15B*, and *MUSTN1*—were assessed using the ABScript II One Step SYBR Green RT-qPCR Kit (ABclonal, Woburn, Massachusetts, USA; Cat. No. RK20404). Primer sequences used for RT-qPCR analysis are provided in Supplementary Table S5. *EVA1C* was also considered for RT-qPCR evaluation because it remained significant after FDR correction in the RNA-seq analysis; however, despite testing alternative primer pairs, a reliable and specific amplification profile could not be achieved, and *EVA1C* was therefore excluded from the RT-qPCR panel.

RT-qPCR reactions were performed on a Rotor-Gene Q real-time PCR system (Qiagen, Hilden, Germany) in a final reaction volume of 20 µL using 2 µL of total RNA (approximately 60 ng/µL; approximately 120 ng total RNA per reaction) as template, according to the manufacturer’s instructions. Reverse transcription was performed at 42 °C for 5 min, followed by pre-denaturation at 95 °C for 1 min and 40 amplification cycles of 95 °C for 5 s and 60 °C for 30 s. Amplification specificity was assessed by melting-curve analysis. All RT-qPCR reactions were performed in technical duplicate, and mean Ct values were used for subsequent analyses. Gene expression levels were normalized to *ACTB* using the ΔCt method. Lower ΔCt values indicate higher relative gene expression. Relative expression changes between patients with MetS and healthy controls were calculated using the 2^−ΔΔCt method, with the control group used as the calibrator.

### 4.5. Statistical Analysis

Baseline continuous variables were summarized as median and interquartile range and compared between MetS and control groups using the Mann–Whitney U test. Categorical variables were presented as n (%) and compared using Fisher’s exact test.

RNA-seq differential expression analysis was performed in R using DESeq2 based on raw gene count data. Differential expression was evaluated using log2 fold-change values and corresponding *p*-values, and multiple testing was controlled using the Benjamini–Hochberg false discovery rate (FDR) method. Genes with nominal *p* < 0.05 were considered in the exploratory analysis, whereas FDR-adjusted *p* < 0.05 was considered statistically significant after correction for multiple testing. Volcano plots and heatmaps were generated in R to visualize differential expression patterns between patients with MetS and healthy controls.

Statistical analyses of RT-qPCR data were performed using IBM SPSS Statistics for Windows, version 26.0 (IBM Corp., Armonk, NY, USA). Gene expression analyses were based on normalized ΔCt values. Because lower ΔCt values indicate higher relative gene expression, the direction of expression differences was interpreted accordingly. Comparisons between patients with MetS and healthy controls were performed using the Mann–Whitney U test. Relative expression changes were expressed as fold changes calculated using the 2^−ΔΔCt method. For the fold-change estimates displayed in Figure 3 and 95% confidence intervals were calculated using bias-corrected and accelerated (BCa) nonparametric bootstrap resampling of individual ΔCt values (10,000 replicates). For each bootstrap sample, the between-group ΔΔCt was calculated and back-transformed using the 2^−ΔΔCt method. These confidence intervals were used to quantify the uncertainty around the fold-change estimates, whereas statistical inference for between-group comparisons was based on the Mann–Whitney U test performed on individual ΔCt values. Multiple testing across the six candidate genes was controlled using the Benjamini–Hochberg FDR method.

Associations between candidate-gene expression and continuous clinical variables, including body mass index, waist circumference, fasting glucose, HbA1c, HDL-C, and triglyceride levels, were assessed using Spearman rank correlation. Hypertension and ischemic heart disease status were analyzed using the Mann–Whitney U test. For these exploratory clinical analyses, Benjamini–Hochberg FDR correction was applied across the full set of gene–phenotype association tests involving all candidate genes and evaluated clinical variables. A two-sided *p*-value < 0.05 was considered nominally significant, and FDR-adjusted *p* < 0.05 was considered statistically significant after correction for multiple testing.

## 5. Conclusions

This study identified peripheral blood transcriptional signals associated with MetS through an RNA-seq discovery approach followed by RT-qPCR evaluation in a larger independent cohort. FKBP1B showed the strongest evidence, remaining significant after transcriptome-wide FDR correction and demonstrating concordant upregulation by RT-qPCR. ARVCF and NAA40 were nominal RNA-seq findings that also showed significantly increased expression in the RT-qPCR cohort, whereas PLIN2, ALOX15B, and MUSTN1 were not significantly different between groups. Exploratory analyses did not identify robust associations between candidate-gene expression and the evaluated metabolic or cardiovascular clinical parameters after correction for multiple testing. Further studies in independent cohorts and mechanistic models are needed to clarify the biological and clinical significance of these findings.

## Data Availability

All data and materials supporting the results or analyses presented in this study are available upon reasonable request.

## Supplementary Materials

The following supporting information can be downloaded at https://www.mdpi.com/article/10.3390/ijms27219564/s1.
