# Supplementary material for: Transcriptomic Alterations in Peripheral Blood of Patients with Metabolic Syndrome
Source: Int J Mol Sci. 2026 Oct 26;27(21):9564. doi: 10.3390/ijms27219564 (PMC13762570; doi:10.3390/ijms27219564)
Supplement: Supplementary file 1 [file ijms-27-09564-s001.zip › Supplementary Table S1.pdf]

Supplementary Table S1. Baseline demographic and clinical characteristics of the study cohorts

Panel A. RNA-seq discovery cohort

Characteristic	MetS (n=12)	Control (n=10)	p-value
Age, years	56.5 [48.5–61.5]	40.0 [39.0–40.0]	<0.001
Male sex, n (%)	4 (33.3%)	5 (50.0%)	0.666
BMI, kg/m²	32.2 [29.8–34.1]	24.3 [21.7–26.4]	<0.001
Waist circumference, cm	103.0 [100.5–104.8]	88.0 [74.0–90.0]	<0.001
Fasting glucose, mg/dL	117.0 [106.0–154.0]	85.0 [78.5–87.0]	<0.001
HbA1c, %	6.3 [5.8–7.7]	5.2 [4.8–5.7]	0.004
Triglycerides, mg/dL	165.0 [112.2–183.3]	87.3 [66.1–110.6]	0.005
HDL-C, mg/dL	47.9 [40.8–53.0]	48.4 [41.4–60.1]	0.598
LDL-C, mg/dL	133.0 [113.2–157.2]	103.8 [88.2–118.8]	0.032
Total cholesterol, mg/dL	211.0 [181.5–234.0]	161.7 [149.1–185.4]	0.038
Hypertension, n (%)	12 (100.0%)	0 (0.0%)	<0.001
Ischemic heart disease, n (%)	1 (8.3%)	0 (0.0%)	1.000
Antidiabetic medication, n (%)	8 (66.7)	0 (0.0)	0.002
Antihypertensive medication, n (%)	12 (100)	0 (0.0)	<0.001
Lipid-lowering medication, n (%)	5 (41.7)	0 (0.0)	0.040

Values are presented as median [interquartile range] or n (%).

Panel B. RT-qPCR evaluation cohort

Characteristic	MetS (n=146)	Control (n=116)	p-value
Age, years	59.0 [50.0–66.0]	37.0 [29.0–51.0]	<0.001
Male sex, n (%)	49 (33.6%)	51 (44.0%)	0.097
BMI, kg/m²	31.1 [28.3–34.3]	23.5 [21.8–26.0]	<0.001
Waist circumference, cm	98.0 [93.0–104.8]	85.0 [75.8–91.2]	<0.001
Fasting glucose, mg/dL	124.5 [110.0–160.0]	87.0 [80.0–93.0]	<0.001
HbA1c, %	6.8 [6.2–8.3]	5.3 [5.1–5.7]	<0.001
Triglycerides, mg/dL	172.0 [129.7–226.0]	90.3 [61.0–131.4]	<0.001
HDL-C, mg/dL	43.0 [38.0–51.0]	51.4 [42.2–65.6]	<0.001
LDL-C, mg/dL	122.0 [95.5–155.0]	106.0 [92.8–125.2]	0.001
Total cholesterol, mg/dL	198.0 [167.0–234.0]	176.0 [156.8–203.4]	<0.001
Hypertension, n (%)	120 (82.8%)	0 (0.0%)	<0.001
Ischemic heart disease, n (%)	31 (21.7%)	0 (0.0%)	<0.001
Antidiabetic medication, n (%)	125 (85.6)	0 (0.0%)	<0.001
Antihypertensive medication, n (%)	112 (76.7)	0 (0.0%)	<0.001
Lipid-lowering medication, n (%)	92 (63.0)	0 (0.0%)	<0.001

Values are presented as median [interquartile range] or n (%).BMI, body mass index; HDL-C, high-density lipoprotein cholesterol; LDL-C, low-density lipoprotein cholesterol.
