# Supplementary material for: Transcriptomic Alterations in Peripheral Blood of Patients with Metabolic Syndrome
Source: Int J Mol Sci. 2026 Oct 26;27(21):9564. doi: 10.3390/ijms27219564 (PMC13762570; doi:10.3390/ijms27219564)
Supplement: Supplementary file 1 [file ijms-27-09564-s001.zip › Supplementary Table S2.pdf]

Supplementary Table S2. RNA-seq characteristics of candidate genes selected for RT-qPCR evaluation.

Gene	Linear fold change	log2 fold change	Nominal p-value	FDR-adjusted p-value	Rank within direction*
FKBP1B	2.0695	1.049	8.27×10^{-7}	0.0194	1st upregulated
PLIN2	1.4153	0.501	1.33×10^{-5}	0.0785	3rd upregulated
ARVCF	1.5864	0.666	1.35×10^{-4}	0.343	11th upregulated
NAA40	1.3112	0.391	3.11×10^{-4}	0.408	18th upregulated
ALOX15B	0.5476	−0.869	1.49×10^{-3}	0.730	10th downregulated
MUSTN1	0.4062	−1.300	1.63×10^{-3}	0.748	13th downregulated
