# Supplementary material for: Transcriptomic Alterations in Peripheral Blood of Patients with Metabolic Syndrome
Source: Int J Mol Sci. 2026 Oct 26;27(21):9564. doi: 10.3390/ijms27219564 (PMC13762570; doi:10.3390/ijms27219564)
Supplement: Supplementary file 1 [file ijms-27-09564-s001.zip › Supplementary Table S3.pdf]

Supplementary Table S3. Spearman correlations between candidate-gene expression and continuous clinical variables in patients with metabolic syndrome.

Clinical variable	Gene	Spearman ρ	p	FDR-Adj. p
**BMI**	*FKBP1B*	0.004	0.967	0.997
	PLIN2	-0.014	0.873	0.997
	ARVCF	-0.110	0.187	0.997
	NAA40	-0.110	0.186	0.997
	ALOX15B	-0.043	0.608	0.997
	MUSTN1	-0.046	0.578	0.997
**Waist circumference**	*FKBP1B*	0.037	0.668	0.997
	PLIN2	-0.102	0.240	0.997
	ARVCF	-0.004	0.959	0.997
	NAA40	-0.062	0.461	0.997
	ALOX15B	-0.005	0.949	0.997
	MUSTN1	-0.058	0.483	0.997
**Fasting glucose**	*FKBP1B*	0.070	0.416	0.997
	PLIN2	-0.096	0.273	0.997
	ARVCF	-0.017	0.840	0.997
	NAA40	0.047	0.575	0.997
	ALOX15B	0.097	0.245	0.997
	MUSTN1	0.003	0.967	0.997
**HbA1c**	*FKBP1B*	0.128	0.141	0.997
	PLIN2	-0.052	0.561	0.997
	ARVCF	-0.074	0.380	0.997
	NAA40	0.016	0.847	0.997
	ALOX15B	0.107	0.210	0.997
	MUSTN1	0.037	0.662	0.997
**HDL-C**	*FKBP1B*	-0.187	**0.032**	0.997
	PLIN2	0.068	0.452	0.997
	ARVCF	-0.078	0.359	0.997
	NAA40	-0.131	0.125	0.997
	ALOX15B	-0.008	0.930	0.997
	MUSTN1	-0.051	0.554	0.997
**Triglycerides**	*FKBP1B*	0.074	0.393	0.997
	PLIN2	-0.079	0.374	0.997
	ARVCF	-0.040	0.637	0.997
	NAA40	-0.052	0.537	0.997
	ALOX15B	0.023	0.782	0.997
	MUSTN1	0.000	0.997	0.997

Analyses were performed using ΔCt values. Higher ΔCt values indicate lower relative gene expression. ***Benjamini–Hochberg FDR correction was applied across the full set of gene–phenotype association tests involving all candidate genes and evaluated clinical variables.***
