# Supplementary material for: Transcriptomic Alterations in Peripheral Blood of Patients with Metabolic Syndrome
Source: Int J Mol Sci. 2026 Oct 26;27(21):9564. doi: 10.3390/ijms27219564 (PMC13762570; doi:10.3390/ijms27219564)
Supplement: Supplementary file 1 [file ijms-27-09564-s001.zip › Supplementary Table S4.pdf]

Supplementary Table S4. Associations between candidate-gene expression and binary cardiovascular clinical characteristics.

Clinical variable	Gene	Median ΔCt (+)	Median ΔCt (−)	p	FDR- Adj p
**Hypertension**	*FKBP1B*	5.98	6.32	0.921	0.958
	PLIN2	8.73	8.75	0.864	0.958
	ARVCF	2.79	2.84	0.848	0.958
	NAA40	3.24	2.81	0.334	0.958
	ALOX15B	5.24	5.07	0.103	0.619
	MUSTN1	2.09	2.22	0.958	0.958
**Ischemic heart disease**	*FKBP1B*	5.97	6.01	0.989	0.989
	PLIN2	8.37	8.76	0.598	0.989
	ARVCF	2.64	2.85	0.897	0.989
	NAA40	3.43	3.13	0.261	0.989
	ALOX15B	5.24	5.16	0.976	0.989
	MUSTN1	1.96	2.11	0.881	0.989
