# Supplementary material for: Transcriptomic Alterations in Peripheral Blood of Patients with Metabolic Syndrome
Source: Int J Mol Sci. 2026 Oct 26;27(21):9564. doi: 10.3390/ijms27219564 (PMC13762570; doi:10.3390/ijms27219564)
Supplement: Supplementary file 1 [file ijms-27-09564-s001.zip › Supplementary Table S5.pdf]

Supplementary Table S5. Primer sequences used for RT-qPCR analysis of candidate genes.

Gene	Forward primer (5′→3′)	Reverse primer (5′→3′)
FKBP1B	GGCAAACAGGAAGTCATCAAAGG	TGGCTCCATATGCCACATCAGG
PLIN2	TTGCAGTTGCCAATACCTATGC	CCAGTCACAGTAGTCGTCACA
ARVCF	GCAGAAAGATGGTTGGACCAAGG	CAAGGCTCTTGTCCACCAGTGG
NAA40	ATGTAAGCGAGTGTCTGGACT	TGGTTTGCATATTCGTTTTGGTC
ALOX15B	GATCTTCAACTTCCGGAGGAC	ACTGGGAGGCGAAGAAGG
MUSTN1	ATGCGAGAGTGTGAGCAAGCT	TTCTCAGCCGAAGACACTCTTGT
ACTB	CACCATTGGCAATGAGCGGTTC	AGGTCTTTGCGGATGTCCACGT
